# Evaluation of the General Population's Knowledge Concerning Liver Health: A Cross-Sectional Study

**DOI:** 10.7759/cureus.54162

**Published:** 2024-02-14

**Authors:** Hidar Alibrahim, Haidara Bohsas, Sarya Swed, Khaled Albakri, Yossef H AbdeQadir, Sara Ramadan, Lazaward Kazan, Heba Haj Saleh, Fatema Ali Asgar Tashrifwala, Mohamad Al Ibrahim, Sabine Tayfour, Touka Abo Alsel, Abdullah Alnehlawi, Ubaid Khan, Ashraf N.B. Boktor, Ibrahim Elbialy, Hekmieh Manad, Reem Rizk Abazid, Wael Hafez

**Affiliations:** 1 Internal Medicine, Aleppo University, Aleppo, SYR; 2 Faculty of Medicine, Aleppo University, Aleppo, SYR; 3 Medicine, Aleppo University, Aleppo, SYR; 4 Faculty of Medicine, The Hashemite University, Zarqa, JOR; 5 General Internal Medicine, Faculty of Medicine, Alexandria University, Alexandria, EGY; 6 Research, International Medical Students' Research Association, Cairo, EGY; 7 Faculty of Medicine, Alexandria University, Alexandria, EGY; 8 Faculty of Medicine, Altinbas University, Istanbul, TUR; 9 Research and Discovery, Stamford Health, Stamford, USA; 10 Biotechnology Engineering, Faculty of Technical Engineering, Aleppo University, Aleppo, SYR; 11 Faculty of Medicine, Damascus University, Damascus, SYR; 12 Community Medicine, King Edward Medical University, Lahore, PAK; 13 Family Medicine, LLH Hospital, Abu Dhabi, ARE; 14 Internal Medicine Emergency, Burjeel Hospital, Abu Dhabi, ARE; 15 Internal Medicine, Mediclinic Hospital, Abu Dhabi, ARE; 16 Obstetrics and Gynaecology, NMC Royal Hospital, Abu Dhabi, ARE; 17 Internal Medicine, NMC Royal Hospital, Abu Dhabi, ARE; 18 Internal Medicine, National Research Centre, Cairo, EGY

**Keywords:** evaluation, syria, observational cross-sectional study, liver health, knowledge

## Abstract

Introduction: Liver disease is among the leading causes of global mortality and morbidity. Given their substantial impact on public health, raising awareness about liver diseases is paramount for their prevention and effective management. This study aimed to evaluate the knowledge, awareness, attitudes, and behaviors of Syrians regarding liver health, chronic liver disorders, and their associated serious and irreversible complications.

Methods: We conducted a cross-sectional study encompassing the adult Syrian population between August 25 and September 29, 2023, excluding non-Syrians and individuals below the age of 18 years. A validated questionnaire, adapted from a previous study, was employed, consisting of 31 questions that covered topics related to knowledge and awareness of liver health and diseases (3-point Likert scale), attitudes towards liver screening, diagnosis, and treatment, and awareness of treatment options and vaccination. Statistical analysis including logistic regression was conducted using Statistical Product and Service Solutions (SPSS, version 28; IBM SPSS Statistics for Windows, Armonk, NY), with statistical significance set established at pp-values below 0.05.

Results: This study included 941 participants, with an average age of 26.5 years. While two-thirds of respondents demonstrated awareness of hepatitis B and C as viral diseases (663 (70.4%) and 612 (65.4%), respectively), approximately 66 (7%) were unaware of the potential for hepatitis to induce chronic liver inflammation or lead to liver failure. Over half of the participants were knowledgeable about the non-genetic nature of hepatitis B and C, and 579 (61.7%) were informed about the transmission risks associated with these infections. The most common reason cited for not participating in health screening tests was the perception of being in good health (219, 77.4%), and prescription medication was the most frequently sought treatment for hepatitis (543, 83.9%). Bivariate analysis revealed correlations between participant knowledge and sex, socioeconomic status, educational level, and occupation (P < 0.05). Similarly, the study identified significant associations between participant attitudes and age, gender, economic status, job, and educational level (P < 0.05). Moreover, the multivariate analysis demonstrated that gender, occupation, and educational level significantly influenced both participants’ knowledge and attitudes. Specifically, males exhibited lower knowledge and less favorable attitudes than females (P = 0.041 and P < 0.001, respectively).

Conclusion: The Syrian population possessed moderate knowledge of liver health and liver disorders. To bridge this knowledge gap and enhance preventive measures, it is recommended that additional health programs and awareness initiatives be implemented, involving healthcare providers and leveraging their expertise.

## Introduction

The liver, a crucial organ responsible for various physiological functions such as nutrient metabolism, detoxification, and bile production, plays a vital role in maintaining overall health [1].

Globally, liver diseases contribute significantly to mortality and morbidity, resulting in approximately two million deaths annually. This includes one million deaths from cirrhosis complications and another million deaths from viral hepatitis and hepatocellular carcinoma (HCC) [2,3]. Cirrhosis, representing the advanced stage of liver scarring due to persistent inflammation, is associated with severe complications, such as portal hypertension, liver failure, and increased mortality [4-6]. Notably, a considerable number of patients or individuals with cirrhosis may remain asymptomatic during the compensated stage [7].

Regionally, the prevalence of liver diseases that lead to cirrhosis varies. In developed countries, common causes include the hepatitis C virus (HCV), alcoholic liver disease, and nonalcoholic steatohepatitis, whereas, in developing countries, hepatitis B and C are the primary contributors to cirrhosis [8].

NASH, a liver condition characterized by lipid accumulation in hepatocytes, results in lipotoxicity and cellular damage. It predominantly affects individuals with obesity or metabolic disorders such as diabetes, insulin resistance, or lipid dysregulation. The uncontrolled inflammation in NASH can progress into fibrosis and cirrhosis [9].

Among the hepatitis viruses, hepatitis B, C, and D are associated with chronic liver diseases, cirrhosis, and liver cancer. Globally, hepatitis B and C had a prevalence of 3.9% (292 million) and 1% (71 million), respectively, in 2015-2016. Notably, half of all hepatitis B cases occur in Nigeria, China, India, and Indonesia. Chronic hepatitis C infection may lead to cirrhosis in 10%-20% of cases and HCC in 1-5%. Cirrhosis significantly elevates the annual HCC incidence up to 5%-7% [10,11].

The New England Journal of Medicine suggested crucial steps upon the cirrhosis diagnosis. These include discontinuing harmful substances such as medications, alcohol, and certain drugs. Blood pressure monitoring, esophageal varices screening, and biannual HCC screenings are essential. With a mean arterial pressure of 82 mm Hg, antihypertensive drugs should be discontinued. If deemed appropriate, liver transplantation evaluation should be initiated [12].

Decompensated cirrhosis poses a 9.7-fold higher mortality risk than that of the general population. Those with compensated cirrhosis have an average life expectancy of 10-13 years, while individuals with decompensated cirrhosis are expected to survive for only two years [12].

Given the often-asymptomatic progression of liver diseases to advanced stages, creating awareness becomes crucial for effective prevention and management [13]. Strategies such as promoting healthy habits, encouraging hepatitis virus vaccination, and avoiding risky behaviors are proven effective in preventing liver diseases [14]. Early detection through screening is equally vital for preventing disease progression and reducing the risk of complications [15].

Despite advances in the understanding of liver function and the pathophysiology of chronic liver disorders globally, liver diseases continue to significantly impact public health [1]. Recognizing this ongoing challenge, further research and the implementation of preventive measures are imperative to improve patient prognosis and enhance their quality of life. This research specifically aims to evaluate the knowledge, awareness, attitudes, and behaviors of Syrians concerning liver health, chronic liver disorders, and related irreversible complications. This study is poised to serve as a valuable reference for future research and prospective public health initiatives, focusing on screening and safeguarding the population.

## Materials and methods

Study design and setting

This cross-sectional study was conducted among the adult Syrian population from August 25 to September 29, 2023. The participants were restricted to Syrian nationals aged 18 years and above. The primary objective was to assess the knowledge, attitudes, and practices related to liver health, chronic liver diseases, their impact on human health, and preventive measures. The study utilized a questionnaire adapted from a previously validated scale [16], with our analysis relying on the same cut-off points. The questionnaire was meticulously translated into Arabic by healthcare professionals proficient in multiple languages to ensure accessibility to the target population. Data collection employed a combination of convenience and snowball sampling methods, distributing the questionnaire both online via Google Forms on various social media platforms and in print for in-person distribution.

Sample size calculation

To determine the appropriate sample size, we referred to World Bank statistics indicating an expected Syrian population of 18,275,704 people in 2021 [17]. Assuming a percentage of 50%, a margin of error of 5%, and a confidence level of 95%, we calculated a minimum sample size of 385 using Calculator.net (www.calculator.net/) [18].

Measures

The questionnaire consisted of three main sections, comprising a total of 31 questions, preceded by eight questions aimed at gathering sociodemographic information from participants. The first section, with 15 questions, focused on assessing the population's awareness of liver health and related disorders. The second section, composed of 13 questions, delved into attitudes regarding liver screening, diagnostic procedures, and the treatment of liver diseases. The third section involved three questions designed to gauge respondents' attitudes toward various local and medical remedies for liver ailments, as well as their awareness of the hepatitis B virus (HBV) vaccine.

Sociodemographic characteristics

This section comprised eight questions, seven of which sought general information about participants, including age, gender, educational status, financial situation, employment status, place of residence, and marital status. The eighth question aimed to ascertain whether the participants had any chronic diseases.

Knowledge and awareness of liver health and diseases

The questionnaire's second section contained 15 questions to assess general knowledge about liver health and related disorders. Respondents used a 3-point Likert scale (agree/disagree/not sure) to answer questions covering various aspects of liver function, methods for maintaining liver health, liver-related disorders, interpretation of elevated liver enzyme levels, hepatitis B and C, and transmission routes. Questions also addressed whether participants associated with certain health issues (such as fibrosis, hypertension, dementia, diabetes, etc.) have liver disorders and whether they were familiar with all types of viral hepatitis. The participants were asked to self-assess their knowledge of hepatitis using a 5-point Likert scale. Finally, the participants were presented with five straightforward questions concerning the stages of liver scarring and the potential for hepatitis to lead to cirrhosis, liver failure, and cancer.

Attitude toward liver screening, diagnosis, and treatment of liver diseases

Participants were asked to rate their willingness to undergo hepatitis screening in various scenarios on a 7-point Likert scale. Additionally, they were queried about their most recent medical check-up, whether they included liver screening tests, whether they had received a diagnosis of hepatitis or another liver condition, and if they were considering treatment, and, if so, their reasons for accepting or declining treatment.

Attitude and awareness toward treatment and vaccination

This section featured three questions designed to assess participants' understanding of the nationally implemented HBV immunization program and their preferences for different treatment modalities for liver conditions, including traditional medicine, over-the-counter medications, or doctor-prescribed treatments.

Pilot study

Before administering the questionnaire to the main research population, a pilot study involving 50 Syrians was conducted to ensure the clarity, accuracy, and comprehensibility of the questionnaire's questions. Based on their responses and feedback, the questionnaires were adjusted accordingly. Furthermore, the reliability and validity of the questionnaire were re-verified by calculating Cronbach's alpha values, which ranged from 0.712 to 0.861.

Ethical considerations

Ethical approval for this study was obtained from the Aleppo University Ethics Committee (IRB-98/76/L9), and all procedures adhered to the Declaration of Helsinki. The participants were presented with a consent question at the outset of the survey. Additionally, essential information about the study's objectives, the research team, participants' right to withdraw at any point, and data confidentiality was provided. The survey took participants between 20 and 30 minutes to complete, and all responses were securely documented and stored in a protected database.

Statistical analysis

Descriptive statistics were employed to summarize participants' demographic profiles, with categorical data presented as frequencies and percentages, and continuous data represented as means and standard deviations. Relevant statistical tests, including chi-square tests and t-tests, were used to evaluate and compare knowledge, attitudes, and confidence scores, when applicable. The analysis was conducted using Statistical Product and Service Solutions (SPSS, version 28; IBM SPSS Statistics for Windows, Armonk, NY), with statistical significance set established at pp-values below 0.05. Multivariate logistic regression analysis was also conducted to explore the combined influence of multiple independent variables on binary or categorical outcome variables related to knowledge and attitudes concerning liver health management, allowing for a more comprehensive understanding of the relationships between the predictor variables and the final outcomes.

## Results

Demographic profile of the study participants

The study included 941 participants between the ages of 17 and 78 years, with a mean age of 26.5 (SD = 10.2). More than half of the participants (546, 58%) were female, and 590 (62.7 %) were students. Approximately three-fourths of respondents (727, 77.3%) were city dwellers. Regarding social status, 729 (77.5%) of the study population were single, while 196 (20.8%) of them were married. The majority of the participants had a university degree (737, 78.3%), whereas 14 (1.5%) of them had attained middle school, and six (0.6%) had no education. The vast majority of them (845, 89.8%) did not report having any chronic conditions. Additionally, 825 (87.7%) reported good or average economic status, whereas 48 (5.1%) reported low financial status. One hundred seventy-five (18.6%) of them were privately employed, and 74 (7.9%) were unemployed (Table 1).

**Table 1:** Participants’ socioeconomic characteristics.

Variables	Frequency (Percentage, %)
Age	
Mean (SD)	26.5 (10.2)
Range	17.0-78.0
Gender	
Female	546 (58.0%)
Male	395 (42.0%)
Living site	
Rural area	214 (22.7%)
Cities	727 (77.3%)
Social status	
Widow	9 (1.0%)
Single	729 (77.5%)
Married	196 (20.8%)
Divorced	7 (0.7%)
Educational level	
Primary school	4 (0.4%)
Middle school	14 (1.5%)
High school	122 (13.0%)
Diploma	51 (5.4%)
University education	737 (78.3%)
Postgraduate	7 (0.7%)
There is no education	6 (0.6%)
Job	
Private work	175 (18.6%)
Public sector	84 (8.9%)
Student	590 (62.7%)
Unemployment	74 (7.9%)
Retired	18 (1.9%)
Economic status	
Low	48 (5.1%)
Average	431 (45.8%)
Good	394 (41.9%)
Excellent	68 (7.2%)
History of chronic disease	
No	845 (89.8%)
Yes	96 (10.2%)

Awareness of hepatitis B and C: a mixed understanding

The study found that approximately two-thirds of participants were aware that hepatitis B and C are viral illnesses (645 (68.5%) and 612 (65%), respectively); however, almost 57% of them reported that hepatitis B and C are bacterial infections. More than half of the study population (>50%) were aware that hepatitis B and C may induce chronic liver inflammation or might cause liver failure. Moreover, 685 (72.8%) of the respondents agreed that hepatitis B could be prevented by vaccination, while 362 (38.5%) of them contended that hepatitis C cannot be prevented through vaccination. Furthermore, 511 (54.3%) and 573 (60.9%) of the respondents acknowledged that hepatitis B and C are not genetic illnesses, respectively. Approximately 6% of the participants did not recognize that hepatitis B and C increased the risk of developing liver cirrhosis and cancer. At a similar rate, the participants had responded unsure that these diseases were not airborne (hepatitis B: 244 (25.9%); hepatitis C: 269 (28.6%); Table 2).

**Table 2:** Knowledge of the features of hepatitis B and hepatitis C.

Variables	Frequency (Percentage, %)	
	Hepatitis B	Hepatitis C
Question (correct response)		
Is a bacterial infection (disagree)		
Agree	151 (16.0%)	155 (16.5%)
Do not agree	579 (61.5%)	532 (56.5%)
Not sure	211 (22.4%)	254 (27.0%)
Is a viral infection (agree)		
Agree	645 (68.5%)	612 (65.0%)
Do not agree	102 (10.8%)	95 (10.1%)
Not sure	194 (20.6%)	234 (24.9%)
Can cause chronic inflammation of the liver (agree)		
Agree	663 (70.5%)	625 (66.4%)
Do not agree	47 (5.0%)	75 (8.0%)
Not sure	231 (24.5%)	241 (25.6%)
Can cause liver failure (agree)		
Agree	663 (70.5%)	642 (68.2%)
Do not agree	72 (7.7%)	66 (7.0%)
Not sure	206 (21.9%)	233 (24.8%)
Can be prevented by vaccination (agree for hepatitis B; disagree for hepatitis C)		
Agree	685 (72.8%)	362 (38.5%)
Do not agree	60 (6.4%)	267 (28.4%)
Not sure	196 (20.8%)	312 (33.2%)
Is airborne (disagree)		
Agree	119 (12.6%)	139 (14.8%)
Do not agree	578 (61.4%)	533 (56.6%)
Not sure	244 (25.9%)	269 (28.6%)
Is hereditary (disagree)		
Agree	121 (12.9%)	148 (15.7%)
Do not agree	573 (60.9%)	511 (54.3%)
Not sure	247 (26.2%)	282 (30.0%)
Increases the risk of the development of liver cirrhosis and cancer (agree)		
Agree	671 (71.3%)	634 (67.4%)
Do not agree	58 (6.2%)	62 (6.6%)
Not sure	212 (22.5%)	245 (26.0%)

Knowledge about transmission risks of hepatitis B and C

The majority of respondents reported that hepatitis B and C could be transmitted through various means, including sexual intercourse (581, 61.7%), contact with blood such as through an open wound (679, 72.2%), and by sharing non-sterile needles or experiencing needlestick injuries (770, 81.8%). A notable 587 (62.4%) believed that these diseases cannot be transmitted by touching an infected person. Additionally, almost half of the study population agreed that transmission through the fecal-oral route was unlikely. Regarding the transmission of hepatitis B and C from a pregnant mother to her baby during birth, 185 (19.7%) of participants were uncertain. Meanwhile, 119 (12.6%) and 406 (43.1%) of respondents rejected the idea that hepatitis B and C can be transmitted via sharing razors or toothbrushes and by receiving tattoos or body piercing from settings with poor infection control standards, respectively. On the other hand, 339 (36%) reported that transmission could occur by dining together with an infected person. Three-quarters of the participants concurred that hepatitis B and C cannot be transferred by eating contaminated or raw seafood, such as shellfish (714, 75.9%; Table 3).

**Table 3:** Knowledge about the transmission risks of hepatitis B and C.

Variables	Frequency (Percentage, %)
By touching an infected person (disagree)	
Agree	218 (23.2%)
Do not agree	587 (62.4%)
Not sure	136 (14.5%)
Through sexual intercourse (agree)	
Agree	581 (61.7%)
Do not agree	194 (20.6%)
Not sure	166 (17.6%)
Through blood, for example, contact with an open wound (agree)	
Agree	679 (72.2%)
Do not agree	127 (13.5%)
Not sure	135 (14.3%)
By sharing nonsterile needles or through needlestick injuries (agree)	
Agree	770 (81.8%)
Do not agree	71 (7.5%)
Not sure	100 (10.6%)
Fecal–oral route usually through contaminated food, for example, an infected person forgets to properly wash hands after using the toilet and contaminate the food (disagree)	
Agree	474 (50.4%)
Do not agree	318 (33.8%)
Not sure	149 (15.8%)
From pregnant mother to her baby at birth (agree)	
Agree	654 (69.5%)
Do not agree	102 (10.8%)
Not sure	185 (19.7%)
By sharing of razors and toothbrushes (agree)	
Agree	682 (72.5%)
Do not agree	119 (12.6%)
Not sure	140 (14.9%)
By receiving tattoos and body piercings from settings with poor infection control standards (agree)	
Agree	284 (30.2%)
Do not agree	406 (43.1%)
Not sure	251 (26.7%)
By eating contaminated or raw seafood, for example, shellfish (disagree)	
Agree	714 (75.9%)
Do not agree	84 (8.9%)
Not sure	143 (15.2%)
Having received blood (products) before around 1990s (agree)	
Agree	700 (74.4%)
Do not agree	69 (7.3%)
Not sure	172 (18.3%)
Having received long-term kidney dialysis (agree)	
Agree	491 (52.2%)
Do not agree	136 (14.5%)
Not sure	314 (33.4%)
By mosquito bites (disagree)	
Agree	248 (26.4%)
Do not agree	413 (43.9%)
Not sure	280 (29.8%)
By dining together (eg, sharing food) with an infected person (disagree)	
Agree	339 (36.0%)
Do not agree	404 (42.9%)
Not sure	198 (21.0%)

Barriers to health screening and treatment seeking among participants: insights from a Syrian population

The most common reasons given for not attending health screening tests were the lack of need for health screening tests and feeling healthy, with 219 (77.4%) of participants citing this reason, followed by 105 (37%) reported that their doctor did not recommend screening tests, and 99 (35%) reported that screening tests are expensive. Among individuals who had previously been diagnosed with a liver problem, the majority (36, 67.9%) received therapy shortly after diagnosis, whereas a significant proportion (8, 15.1%) never received treatment. The most common answers for not obtaining treatment were "doctor did not prescribe any treatment" (452, 50%), "doctor was not able to clearly explain the treatment plan" (404, 44.7%), and "prescription medicine was not available in our area" (380, 42%). In addition, the respondents indicated that they were uncertain of doctors recommending observation and follow-up without initiating treatments or unable to receive treatment due to a lack of insurance or being underinsured (156 (17.3%) and 127 (14%), respectively). The additional details are provided in Table 4.

**Table 4:** Reasons for not attending health screening or receiving treatment from a hospital or clinic.

Variables	Frequency (Percentage, %)
The reasons that people have given for not attending health screening tests (multiple answers) (N = 283)	
Do not see a reason for going for health screening tests since they feel they are healthy	219 (77.4%)
The doctor did not recommend health screening tests	105 (37.1%)
Health screening tests are expensive	99 (35%)
Health screening tests are not routine	73 (25.8%)
Going for health screening tests is a hassle due to a busy schedule	52 (18.4%)
Health insurance does not cover screening	46 (16.3%)
Fear of discrimination at the workplace or socially if diagnosed with a disease, for example, HIV, cancer, mental illness, hepatitis, etc. during health screening	19 (6.7%)
Thinking of the more recent liver condition you have been diagnosed with, when did you start treating your condition after diagnosis? (N = 53)	
Right after diagnosis	36 (67.9%)
1-2 months	3 (5.7%)
3-6 months	3 (5.7%)
<6 months	3 (5.7%)
Never had a treatment	8 (15.1%)
Others also mentioned that these are the reasons for not receiving treatment from a hospital/clinic. Which of the following statements applies to you? (multiple answers) (N = 904)	
Prescription treatment was too expensive	
Agree	370 (40.9%)
Do not agree	377 (41.7%)
Not sure	157 (17.4%)
Did not believe in Western medicine	
Agree	86 (9.5%)
Do not agree	738 (81.6%)
Not sure	80 (8.8%)
Did not believe that the condition was life-threatening	
Agree	334 (36.9%)
Do not agree	450 (49.8%)
Not sure	120 (13.3%)
Was hesitant because of the side effects	
Agree	243 (26.9%)
Do not agree	542 (60.0%)
Not sure	119 (13.2%)
Was hesitant because it would disrupt normal life	
Agree	244 (27.0%)
Do not agree	546 (60.4%)
Not sure	114 (12.6%)
The doctor did not prescribe any treatment	
Agree	452 (50.0%)
Do not agree	309 (34.2%)
Not sure	143 (15.8%)
The doctor was not able to explain the treatment plan clearly	
Agree	404 (44.7%)
Do not agree	339 (37.5%)
Not sure	161 (17.8%)
Prescription medicine was not available in our area	
Agree	380 (42.0%)
Do not agree	388 (42.9%)
Not sure	136 (15.0%)
Was unable to receive treatment because of lack of insurance or being underinsured	
Agree	321 (35.5%)
Do not agree	456 (50.4%)
Not sure	127 (14.0%)
The doctor recommends observation and follow-up without initiating treatment	
Agree	365 (40.4%)
Do not agree	383 (42.4%)
Not sure	156 (17.3%)

**Figure 1:**
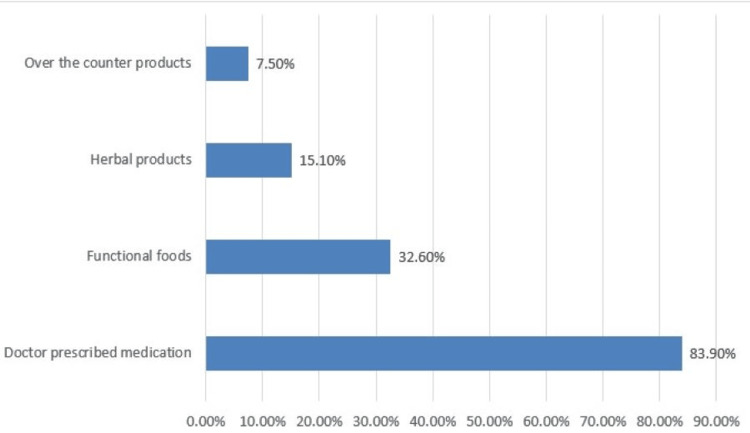
Treatment sought for hepatitis by the respondents (N= 905).

As shown in Figure 1, the most common therapy for hepatitis sought by respondents (83.9%) was a prescription medicine; otherwise, 32.6%, 15.1%, and 7.5% relied on functional food, herbal remedies, and over-the-counter items, respectively.

**Figure 2:**
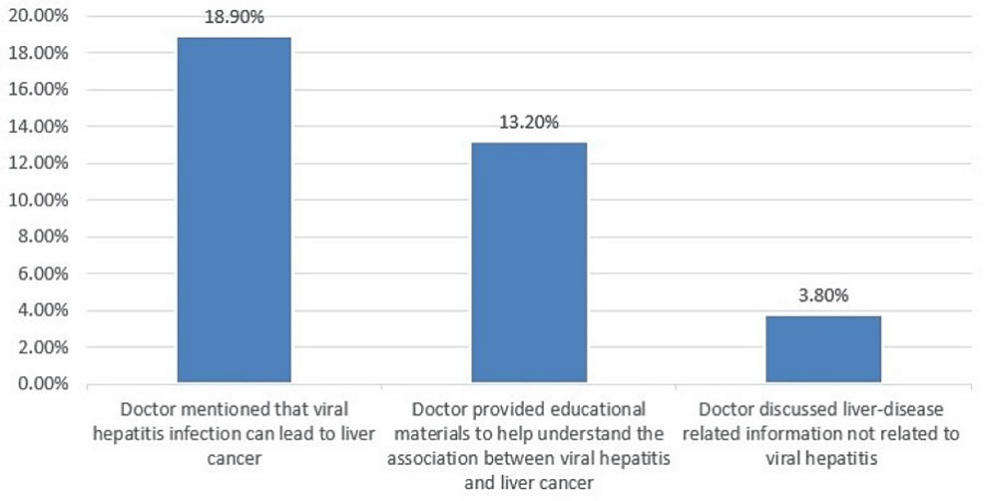
Types of patient-doctor interactions.

Only 18.9% of people afflicted with hepatitis said that their physicians informed them that viral hepatitis infection may develop into live cancer, and 13.2% of them reported their doctors provided educational materials to help understand the association between viral hepatitis and liver cancer (Figure 2).

Demographic correlation of knowledgeability and attitudes towards liver health in the Syrian population

Bivariate analysis revealed a relationship between participant knowledge and gender, social status, educational level, age, and job (P < 0.05). As shown in Table 5, the knowledgeable group had a higher proportion of females (327, 61.9%) compared to the non-knowledgeable group (219, 53.0%). Knowledgeable individuals were more likely to be single (447, 84.7%) compared to non-knowledgeable individuals (282, 68.3%). On the other hand, non-knowledgeable individuals were more likely to be married (121, 29.3%) compared to knowledgeable individuals (75, 14.2%). The participants with a university education showed a higher rate of knowledge than the non-knowledgeable group (282, 68.3%). In contrast, the participants with postgraduate demonstrated lower knowledge than the non-knowledgeable group (1, 0.2%). Approximately, on the knowledgeable group, half of the participants showed a higher proportion to be students compared to the non-knowledgeable group (220, 53%). Additionally, the mean age of the people demonstrated a higher proportion of knowledge than the non-knowledgeable people (49, 11.9%).

**Table 5:** Factors associated with participants’ knowledge. 1: Chi-square test, 2: Mann-Whitney U test

Variables	Frequency (percentage, %)	Frequency (percentage, %)	
	Non-knowledgeable (N=413)	Knowledgeable (N=528)	P value
Gender			0.006^1^
Female	219.0 (53.0%)	327.0 (61.9%)	
Male	194.0 (47.0%)	201.0 (38.1%)	
+Living site			0.647^1^
Rural area	91.0 (22.0%)	123.0 (23.3%)	
Cities	322.0 (78.0%)	405.0 (76.7%)	
Social status			< 0.001^1^
Widow	7.0 (1.7%)	2.0 (0.4%)	
Single	282.0 (68.3%)	447.0 (84.7%)	
Married	121.0 (29.3%)	75.0 (14.2%)	
Divorced	3.0 (0.7%)	4.0 (0.8%)	
Educational level			< 0.001^1^
Primary school	4.0 (1.0%)	0.0 (0.0%)	
Middle school	10.0 (2.4%)	4.0 (0.8%)	
High school	79.0 (19.1%)	43.0 (8.1%)	
Diploma	34.0 (8.2%)	17.0 (3.2%)	
University education	282.0 (68.3%)	455.0 (86.2%)	
Postgraduate	1.0 (0.2%)	6.0 (1.1%)	
There is no education	3.0 (0.7%)	3.0 (0.6%)	
Job			< 0.001^1^
Private work	98.0 (23.7%)	77.0 (14.6%)	
Public sector	33.0 (8.0%)	51.0 (9.7%)	
Student	220.0 (53.3%)	370.0 (70.1%)	
Unemployment	47.0 (11.4%)	27.0 (5.1%)	
Retired	15.0 (3.6%)	3.0 (0.6%)	
Economic status			0.237^1^
Low	25.0 (6.1%)	23.0 (4.4%)	
Average	181.0 (43.8%)	250.0 (47.3%)	
Good	171.0 (41.4%)	223.0 (42.2%)	
Excellent	36.0 (8.7%)	32.0 (6.1%)	
History of chronic disease			0.136^1^
No	364.0 (88.1%)	481.0 (91.1%)	
Yes	49.0 (11.9%)	47.0 (8.9%)	
Age			< 0.001^2^
Mean (SD)	28.0 (11.9)	25.3 (8.4)	

Participants’ attitudes were strongly related to their age, gender, economic status, job, and educational level (P < 0.05). There were significant differences in the distribution of educational levels between the two groups (good and low attitudes). For example, the proportion of individuals with a diploma was higher in the group with a good attitude (543, 83.8%) compared to the other groups (194, 66.2%). The proportion of individuals with a high school education was higher in the group with a low attitude (62, 21.2%) compared to the other group (60, 9.3%). Females had the highest proportion of positive attitudes towards hepatitis (415, 64%) compared to males, who appeared to have the lowest good attitudes towards hepatitis (233, 36%). The study population's mean age proportion in the group with a low attitude towards hepatitis was higher (34, 11.5%) than the group with a good attitude towards hepatitis despite having the same mean age. The individuals with good income status demonstrated good attitudes (314, 48.5%) more than other low-income groups with low attitudes. Retired appeared the lowest good attitude than other job categories with low attitudes (10, 1.5%; Table 6).

**Table 6:** Factors associated with participants’ attitudes. 1: Chi-square test, 2: Mann-Whitney U test

	Frequency (percentage, %)	Frequency (percentage %)	
	Low attitude	Good attitude	
Gender			< 0.001^1^
Female	131.0 (44.7%)	415.0 (64.0%)	
Male	162.0 (55.3%)	233.0 (36.0%)	
Living site			0.818^1^
Rural area	68.0 (23.2%)	146.0 (22.5%)	
City	225.0 (76.8%)	502.0 (77.5%)	
Social status			0.138^1^
Widow	3.0 (1.0%)	6.0 (0.9%)	
Single	215.0 (73.4%)	514.0 (79.3%)	
Married	71.0 (24.2%)	125.0 (19.3%)	
Divorced	4.0 (1.4%)	3.0 (0.5%)	
Educational level			< 0.001^1^
Primary school	8.0 (2.7%)	6.0 (0.9%)	
Middle school	4.0 (1.4%)	0.0 (0.0%)	
High school	62.0 (21.2%)	60.0 (9.3%)	
Diploma	194.0 (66.2%)	543.0 (83.8%)	
University education	20.0 (6.8%)	31.0 (4.8%)	
Postgraduate	3.0 (1.0%)	4.0 (0.6%)	
There is no education	2.0 (0.7%)	4.0 (0.6%)	
Job			0.018^1^
Private work	71.0 (24.2%)	104.0 (16.0%)	
Public sector	27.0 (9.2%)	57.0 (8.8%)	
Student	164.0 (56.0%)	426.0 (65.7%)	
Unemployment	23.0 (7.8%)	51.0 (7.9%)	
Retired	8.0 (2.7%)	10.0 (1.5%)	
Economic status			0.037^1^
Low	129.0 (44.0%)	265.0 (40.9%)	
Average	19.0 (6.5%)	29.0 (4.5%)	
Good	117.0 (39.9%)	314.0 (48.5%)	
Excellent	28.0 (9.6%)	40.0 (6.2%)	
History of chronic disease			0.339^1^
No	259.0 (88.4%)	586.0 (90.4%)	
Yes	34.0 (11.6%)	62.0 (9.6%)	
Age			0.013^2^
Mean (SD)	27.7 (11.5)	25.9 (9.5)	

Table 7 illustrates the results of the multivariate analysis, indicating a significant association between gender and the participants' knowledge and attitudes. Specifically, males demonstrated lower knowledge and a less positive attitude compared to females (OR = 0.74, 95% CI (0.56-0.99), P = 0.041; OR = 0.53, 95% CI (0.39-0.71), P < 0.001, respectively). Additionally, married individuals exhibited lower knowledge levels than their single counterparts (OR = 0.42, 95% CI (0.25-0.68); P < 0.001).

**Table 7:** Determinants of the knowledge and attitudes of the participants.

Variables	OR (95% CI)	P value
Knowledgeability		
Age	1.02 (0.99-1.05)	0.053
Gender		
Female	1	
Male	0.74 (0.56-0.99)	0.041
Social status		
Widow	0.26 (0.038-1.85)	0.18
Single	1	
Married	0.42 (0.25-0.68)	<0.001
Divorced	0.72 (0.14-3.79)	0.69
Educational level		
Primary school	NA	
Middle school	0.32 (0.04-2.65)	0.29
High school	0.28 (0.047-1.65)	0.159
Diploma	0.75 (0.13-4.3)	0.744
University education	0.32 (0.05-2.02)	0.226
Postgraduate	5.35 (0.31-92.75)	0.249
There is no education	1	
Job		
Private work	0.52 (0.34-0.78)	0.002
Public sector	1.17 (0.66-2.07)	0.6
Student	1	
Unemployment	0.48 (0.27-0.87)	0.015
Retired	0.12 (0.025-0.58)	0.008
Attitude		
Age	1 (0.98-1.02)	0.989
Gender		
Female	1	
Male	0.53 (0.39-0.71)	<0.001
Educational level		
Primary school	NA	
Middle school	0.52 (0.07-4.05)	0.528
High school	0.58 (0.1-3.48)	0.55
Diploma	1.52 (0.26-8.94)	0.64
University education	0.91 (0.14-5.78)	0.92
Postgraduate	0.99 (0.09-10.51)	0.99
There is no education	1	
Job		
Private work	0.6 (0.4-0.92)	0.017
Public sector	0.94 (0.52-1.69)	0.824
Student	1	
Unemployment	0.91 (0.49-1.71)	0.778
Retired	0.64 (0.19-2.19)	0.471
Economic status		
Low	1	
Average	0.92 (0.47-1.8)	0.808
Good	1.21 (0.61-2.37)	0.586
Excellent	0.9 (0.39-2.06)	0.799

Job type also emerged as a significant factor associated with both knowledge and attitudes. Unemployed individuals and those working in private jobs demonstrated lower knowledge and attitude levels compared to students, who were used as the reference category (OR = 0.48, 95%CI (0.27-0.87); P = 0.015; OR = 0.52, 95%CI (0.34-0.78); P = 0.002), (OR = 0.91, 95%CI (0.49-1.71); P = 0.77; OR = 0.6, 95%CI (0.4-0.92); P = 0.01).

## Discussion

Hepatitis is a viral infection that causes liver injury and inflammation, leading to a weakened immune system, increased susceptibility to infection, impaired blood filtration, and nutrient processing. It poses a significant global challenge, impacting both morbidity and mortality, and has the potential for outbreaks [19].

This study aimed to assess the knowledge, awareness, attitudes, and understanding of liver health and diseases among the Syrian population. The goal is to establish clear reference points for future public health screening and protection initiatives, as well as for further studies. It is important to note that this research was conducted online, which may have introduced bias toward individuals with higher education and socioeconomic status [20].

The data revealed a reasonably good level of public awareness regarding fundamental aspects of liver virus infections, especially hepatitis B and C. Approximately two-thirds of the respondents correctly identified hepatitis B and C as viral infections rather than bacteria. Additionally, over (50%) of participants demonstrated a clear understanding of how hepatitis can progress to chronic liver inflammation and liver cancer.

However, 362 (38.5%) of the respondents believed that hepatitis C cannot be prevented through vaccination, whereas 685 (72.8%) recognized that vaccination could prevent hepatitis B. Additionally, a small percentage of the study population does not know that hepatitis B and C can increase the risk of developing cancer and liver cirrhosis, with 58 (6.2%) for hepatitis B and 62 (6.6%) for hepatitis C. These findings align with a published study in Thailand, where over (80%) of the participants agreed that hepatitis B and C could lead to liver failure, liver cirrhosis, and cancer. In contrast to our study, almost half of the participants in that study mistakenly considered hepatitis B and C bacterial infections [16]. Another study similar to ours found that more than three-quarters of the participants believed vaccinations could prevent hepatitis B, but 280 (28.9%) mistakenly believed there was a vaccine for hepatitis C [21].

Our results revealed that most participants correctly knew that hepatitis B and C could be transmitted through sexual intercourse, blood, and by sharing non-sterile needles or through needlestick injuries, with percentages of 61.7%, 72.2%, and 81.8%, respectively. In contrast, previous research found low awareness of the transmission of hepatitis B and C, where only 28.3%, 16.3%, and 29.3% of individuals reported that hepatitis B and C could be transferred via contaminated blood, razor blades or nail clippers, and unprotected sex, respectively [22].

Our data revealed prevalent misconceptions about hepatitis transmission, such as the mistaken belief that hepatitis viruses can spread through the fecal-oral route or by consuming contaminated or raw seafood. These findings align with those of prior research from different regions, emphasizing persistent myths regarding hepatitis transmission [23].

Our study found that respondents with an average age of 26.5 years exhibited varying levels of awareness. Younger participants tended to have higher knowledge levels, possibly due to increased access to health-related information on the Internet among this demographic [24]. Similar research in India also identified a correlation between younger age and higher knowledge levels, which was attributed to improved information accessibility for younger individuals [25].

Our bivariate analysis indicated that 327 (61.9%) of females, 447 (84.7%) of single individuals, 75 (14.2%) of married individuals, and 455 (86.2%) with a university education had higher knowledge proportions. This aligns with our study's overall findings, showing that lower educational attainment is associated with lower knowledge of viral hepatitis and its consequences. As education levels increased, knowledge scores also increased. However, in contrast to our study, no association with knowledge was observed based on economic status [26]. A separate cross-sectional study of the Jordanian population revealed a strong correlation between educational level, monthly income, and knowledge about hepatitis B [27].

Notably, individuals who were unemployed or employed in the private sector exhibited lower knowledge levels. This observation may be attributed to European regulations mandating HBV vaccination and proof of immunization for specific job positions [28].

This study identified barriers to participating in health-screening examinations. These barriers include a lack of perceived urgency, infrequent physician recommendations for liver screening, financial constraints, and social stigma and discrimination faced by individuals diagnosed with hepatitis. The stigma surrounding viral hepatitis is a widespread issue and has been linked to low uptake of hepatitis B and C screening. Despite the existence of anti-discrimination laws in many countries, workplace discrimination against individuals with viral hepatitis remains a concern, especially in Southeast and Southern Asia [29]. These outcomes align with prior research, which revealed that obstacles to treatment include disrespect and a lack of consideration from some physicians and nurses, as well as not receiving equal healthcare [30].

Almost two-thirds of the respondents reported seeking treatment upon receiving a diagnosis of hepatitis. However, financial constraints and unavailability of medications in their area were cited as reasons for not seeking treatment. Effective doctor-patient communication and the prescription of appropriate treatment have emerged as critical factors in ensuring treatment adherence. Physicians were identified as the primary source of information for patients, underscoring the importance of education for healthcare providers. A qualitative study in Ireland found that connections with healthcare providers increase their engagement with care [31].

In terms of attitudes and knowledge, married individuals demonstrated higher scores than their single counterparts, while female participants exhibited superior attitudes and knowledge compared to male participants. These observations may be linked to the influence of marital status on health-related awareness and attitude. Married individuals may be more likely to engage in health-related discussions within their households, fostering a shared understanding of diseases and preventive measures. In addition, cultural and societal expectations of family responsibilities may contribute to increased health awareness among married individuals. The observed gender disparity, with females showing higher knowledge and attitudes, could be attributed to the targeted awareness campaigns. Ongoing efforts to educate and empower women on health matters, especially those focusing on women's health, seem to positively impact knowledge levels among females. These findings align with previous research [32] on women's knowledge and health behavior regarding hepatitis B and C, suggesting that continued campaigns to educate and empower women on health matters have a positive impact. Furthermore, awareness campaigns targeting pregnant women and women of reproductive age likely contribute to these results, as they are considered high-risk demographics for mother-to-child disease transmission. The implementation of these strategies holds the promise of facilitating early detection, alleviating the burden of liver diseases in the Syrian community, and enhancing knowledge of liver health among the Syrian population. Ultimately, this leads to a positive attitude and improved behaviors that prevent hepatitis in the population [33].

Initiating public awareness campaigns focused on liver health and chronic liver diseases, and the significance of early detection is paramount. These campaigns encompass the creation of informative brochures, posters, and online resources, all aimed at educating the population about risk factors, symptoms, and available preventative measures.

Conducting community workshops and seminars led by healthcare professionals is another pivotal approach. These events delve into a range of topics, including the vital functions of the liver, the consequences of lifestyle choices on liver health, and the imperative nature of regular check-ups. Interactive sessions play a pivotal role in facilitating a deeper understanding.

Incorporating liver health education into school curricula is a proactive measure. This educational approach not only reaches a younger audience but also instills healthy habits from an early age, ensuring that future generations possess a more comprehensive understanding of liver health.

Collaboration with local healthcare providers, clinics, and hospitals is instrumental in offering liver health screening and consultation. The objective is to motivate individuals to pursue regular check-ups while simultaneously making these services more accessible and cost-effective.

Leveraging diverse media channels, including television, radio, and social media, is an effective strategy for disseminating information on liver health. Consistent featuring stories, interviews, and insights from experts maintains public engagement and awareness of liver health issues.

Establishing support groups for individuals living with liver disease and their families is a compassionate initiative. These groups not only provide emotional support but also impart invaluable information. Additionally, establishing helplines or hotlines where individuals can seek guidance and answer queries about liver health concerns further enhances the support network.

Study limitations and recommendations

This study, the first of its kind in Syria, covered all governorates but had limitations. The exclusion of individuals under 18 years of age may have overlooked the perspectives of teenagers, who may have unique insights into viruses and chronic diseases. The study did not include healthcare professionals, whose viewpoints were valuable. Moreover, the overwhelming presence of single individuals in the sample may not represent families, as intended. To enhance the generalizability of the findings, future research should encompass rural areas and underrepresented populations with lower socioeconomic status. Additionally, including questions about respondents' prior test results or vaccination status could provide a more comprehensive understanding.

## Conclusions

The Syrian population exhibited moderate knowledge about liver health and disorders. Closing the knowledge gap and enhancing prevention measures can be achieved through health programs, awareness campaigns, and the involvement of healthcare providers. Education, training, and promotional initiatives that stress early diagnosis, effective treatment, and preventive measures can significantly contribute to public health.
